# Synthesized evidence for childhood acute lymphoblastic leukemia

**DOI:** 10.3389/fped.2023.1209330

**Published:** 2023-07-24

**Authors:** Felix M. Onyije, Ann Olsson, Liacine Bouaoun, Joachim Schüz

**Affiliations:** Environment and Lifestyle Epidemiology Branch, International Agency for Research on Cancer (IARC/WHO), Lyon, France

**Keywords:** childhood leukemia, risk factors, environmental exposures, pesticides, radon, cesarean delivery

Childhood leukemia is the most common type of cancer among children globally (1). In this study, we evaluated the strength of evidence and magnitude of risk factors for childhood acute lymphoblastic leukemia (ALL) using relevant systematic reviews and pooled analyses that were not part of our previously published umbrella review (1). We also estimated the prevalence in the French population as an example of the relevance of different risk factors. The strength of the association was evaluated using the summary RR/OR values of the various meta-analyses and categorized as very strong (RR > 5), strong (RR > 2), moderate (RR > 1.5), modest (RR > 1.2), and weak (RR > 1). The strength of association, heterogeneity across studies, and number of studies were used to evaluate the strength of evidence. The evidence was categorized into “strong” (consistently strong or very strong risk estimates in quality systematic review and meta-analysis), “some” (consistently moderate risk estimates in quality systematic review and meta-analysis), “little” (consistently low risk estimates), “no” (consistently no association), and “conflicting.” The category of “conflicting” was used when systematic reviews on the same subject came to different conclusions (1). In this article, we provide an update on some of these risk factors strengthening our earlier findings and further promote the prevention of childhood ALL.

During the 70th World Health Assembly, the 2017 WHO cancer resolution adopted a global initiative for cancer prevention and control through an integrated approach (WHA70.12), which focused on reducing premature mortality from non-communicable diseases (NCDs) and achieving universal health coverage. Member states were strongly encouraged to promote the primary prevention of cancers (2). Identifying childhood ALL risk factors, especially modifiable risk factors, is crucial for the success of this campaign.

The first update is on maternal exposure to pesticides during preconception or pregnancy. A recently published systematic review (3) confirmed our previous assessment of convincing evidence of an increased ALL risk (1). In their systematic review and meta-analysis, the authors reported the strongest association in maternal outdoor exposure to pesticides during pregnancy, with more than a twofold increase in effect estimates (RR: 2.51, 95% CI: 1.39–4.55) based on three studies. The effect estimates for insecticides were 1.35 (95% CI: 0.91–2.01) based on four studies, while for herbicides, it was 1.19 (95% CI: 0.92–1.53) based on three studies. Paternal occupational exposure to pesticides at any time of the child's development was associated with childhood leukemia (OR: 1.20, 95% CI: 1.07–1.35) based on five studies. These findings were similar to the pooled summary estimates for general pesticide exposure during childhood and the risk of leukemia (OR: 1.82, 95% CI: 1.07–3.11), and for childhood exposure to insecticides and the risk of ALL (OR: 1.48, 95% CI: 1.17–1.86) based on five and two studies, respectively (Table 1). Some hypotheses suggest that childhood ALL associated with pesticide exposure may result from induced topoisomerase II inhibition leading to misrepaired DNA cleavage and further chromosomal aberrations in hematopoietic stem and/or progenitor cells (9). Greaves had earlier proposed the causal mechanism of childhood ALL to occur due to two genetic hits, with the first hit possibly being introduced by parental exposure to toxic substances during pregnancy (10).

**Table 1:** Recently published environmental risk factors for childhood acute lymphoblastic leukemia.

Authors	Study design^a^	Number of studies	Exposure group	Exposure type/agent	Evidence^b^	Leukemia	Prevalence^c^	RR, 95% CI^d^
Maternal exposure during pregnancy								
Yang et al. (2022) (4)	SR	16	Intrinsic	Cesarean delivery	Little	ALL	High	1.18, 1.07–1.31
Marley et al. (2022) (5)		11		Maternal diabetes	Some	ALL	Moderate	1.46, 1.28–1.67
Karalexi et al. (2021) (3)		3	Pesticides	General—outdoor		ALL	Common	2.51, 1.39–4.55
Karalexi et al. (2021) (3)		4	Pesticides	Occupational		Leukemia	Common	2.08, 1.69–2.56
		3		Herbicides		ALL	Common	1.19, 0.92–1.53
		4		Insecticides		ALL	High	1.35, 0.91–2.01
Paternal exposure at any time (during preconception, pregnancy, or childhood)								
Karalexi et al. (2021) (3)		5	Pesticides	Occupational	Some	Leukemia	Common	1.20, 1.07–1.35
Postnatal exposure								
Karalexi et al. (2021) (3)	SR	5	Pesticides	Any pesticides	Some	Leukemia	High	1.82, 1.07–3.11
		2		Insecticides		ALL		1.48, 1.17–1.86
		3		Any pesticides		ALL	High	2.25, 0.78–6.53
		2		Herbicides		Leukemia	High	1.49, 0.88–2.54
Moon and Yoo (2021) (6)	SR	8	Radiation	Radon	Little	Leukemia	Moderate	1.03, 1.00–1.05
Ngoc et al. (2022) (7)		8					Moderate	1.43, 1.19–1.72
Present article		12				Leukemia	Moderate	1.36, 1.11–1.68
Brabant et al. (2022) (8)	SR	11		Direct MF measurement <0.2 vs. >0.2 µT	Some	Leukemia		1.23, 1.03–1.47
	SR	4		Calculated MF measurement <0.2 vs. >0.2 µT		Leukemia		1.21, 0.64–2.29
	SR	4		Distance between child's home and power lines measurement >200 m vs. <200 m		Leukemia		1.07, 0.71–1.63
	SR			Wire coding low vs. high current configuration		Leukemia		1.87, 1.36–2.56

^a^ SR stands for systematic review.

^b^ Evidence category reflects those in the same rows by exposure type.

^c^ Prevalence for France and sources are different from RR data: high (>20%), common (>10%), moderate (>5%), modest (>2%), and rare (<2%).

^d^ RR also includes OR.

The second update is on childhood exposure to domestic radon and the risk of leukemia. In our previous review, we concluded “conflicting evidence” based on a meta-analysis of cohort studies (two studies; OR = 0.97, 95% CI: 0.81–1.15) and of case–control studies (eight studies; OR = 1.22, 95% CI: 1.01–1.42), with somewhat conflicting results. In recent systematic reviews and meta-analyses, Moon and Yoo (6) reported a summary risk estimate of 1.03 (95% CI: 1.01–1.06) per 100 Bq/m^3^ radon increase based on eight case–control studies with moderate heterogeneity across studies. Ngoc et al. evaluated eight case–control studies, yielding a summary risk estimate of 1.43 (95% CI: 1.19–1.72) (7). As the case–control studies included in those meta-analyses only partly overlapped with our previous review (1), we repeated our meta-analysis with 12 case–control studies, observing an increased summary risk estimate of 1.36 (95% CI: 1.11–1.66), with a heterogeneity of 52.8%, *P*-value = 0.02 (Table 1). Based on this finding, we upgraded radon to “little” evidence.

Further updates are on cesarean delivery and maternal diabetes. The evidence was categorized as “little” for cesarean delivery (OR: 1.18, 95% CI: 1.07–1.31) and “some” for maternal diabetes (OR: 1.46, 95% CI: 1.28–1.67), as reported in our previous review. These findings remained unchanged even after the addition of new studies (4, 5). In addition, with regard to exposure to extremely low-frequency magnetic fields (ELF-MF), the earlier evaluation of “some” evidence remained unchanged even after the inclusion of an additional systematic review (8). The prevalence of the highlighted risk factors in France varied from “common” to “high,” except for electromagnetic field exposure, which is rare (11–16), confirming the importance of risk identification in any primary prevention.
